# Retention in the Austrian opioid agonist treatment system: a national prospective cohort study

**DOI:** 10.1186/s12954-021-00473-9

**Published:** 2021-02-24

**Authors:** Martin Busch, Charlotte Klein, Alfred Uhl, Hans Haltmayer, Maurice Cabanis, Jean Nicolas Westenberg, Marc Vogel, R. Michael Krausz

**Affiliations:** 1 Gesundheit Österreich GmbH (GÖG), Vienna, Austria; 2 Sigmund Freud University, Vienna, Austria; 3 Suchthilfe Wien, Vienna, Austria; 4 Zentrum Für Seelische Gesundheit, Klinikum Stuttgart, Stuttgart, Germany; 5 Department of Psychiatry, University of British Columbia, Vancouver, Canada; 6 Psychiatrische Klinik Münsterlingen, Münsterlingen, Switzerland; 7 Addictions and Concurrent Disorders Research Group, Institute of Mental Health, University of British Columbia, David Strangway Building, 5950 University Boulevard, Vancouver, BC V6T 1Z3 Canada

**Keywords:** Opioid use disorder, Opioid agonist treatment, Treatment retention, Slow-release morphine, National registry

## Abstract

**Background:**

Retention in care is a prerequisite for successful recovery, especially for a chronic condition like opioid dependence. Though retention varies greatly depending on the different substitution medication and treatment model, treatment retention is used as an indicator of treatment quality and effectiveness of care on a system and individual level. To monitor the overall quality of the Austrian opioid agonist treatment (OAT) system and to monitor patient satisfaction within the system, a new online-based registry called “eSuchmittel” was introduced in Austria at the beginning of 2011. The objective of this study is to analyze retention rates within the Austrian treatment system and to identify patient characteristics associated with retention, using data collected by the substitution registry.

**Methods:**

The complete Austrian sample of 4778 registered patients starting treatment between 1.1.2011 to 31.12.2012 were included in the prospective cohort study using data from the Austrian substitution registry. For the statistical analysis, multivariate Cox Regression and Kaplan–Meier survival analysis were used to evaluate retention in treatment.

**Results:**

The retention rate of the total cohort after two years was around 61%. Retention rates were significantly lower for men (exp(B) = .806, 95% CI 0.714–0.908) and significantly higher for patients aged 30 and older (exp(B) = 1.155, 95% CI 1.044–1.279), among patients located in Vienna (exp(B) = 1.439, 95% CI 1.273–1.626) and among patients prescribed oral slow-release morphine (SROM) (exp(B) = 2.141, 95% CI 1.885–2.430).

**Conclusions:**

Average retention in the Austrian system is high in comparison to international retention rates. Nationally, SROM demonstrates higher treatment retention when compared to other available substitution medications. Sociodemographic and regional indicators also contribute to higher retention in care. A systematic monitoring of retention rates within a national registry is an important tool helping to evaluate the quality of care. In this study, the Austrian OAT system proves very high retention in care, an important success criterion.

## Introduction

Opioid agonist treatment (OAT) is the most effective evidence-based treatment strategy for opioid dependence, and contributes to outcomes like psychosocial stabilization, reintegration into society and reduction of drug related mortality [1–5]. Other well-documented outcomes of OAT include reduction of high-risk drug use, reduction of criminal activities and prevention of drug related infectious diseases [6–8].

Overall retention in treatment constitutes a broadly accepted indicator which is used to evaluate the quality of OAT [9–11]. Retention in OAT significantly reduces the risk of all-cause mortality and overdose mortality for those dependent on opioids, whereas poor retention and dropping out of treatment has been shown to increase mortality risk [12]. Additionally, the mortality risk is much higher in the first four weeks of treatment than in the remainder of treatment, and is significantly reduced provided the duration of substitution treatment exceeds one year [12, 13] Further analyses have also shown that patients staying in treatment for a year or longer were nearly five times more likely to have better outcomes, and that length of treatment stay was predicted by higher patient motivation at intake and early program involvement [14]. Positive treatment outcomes such as treatment completion or longer treatment retention have also been related to greater service intensity and quality [15]. These results underline the special importance of OAT retention length towards treatment outcome, as well as the relationship between quality of care, treatment retention, and patient outcome.

A systematic review found a wide range of retention rates in OAT among randomized controlled trials (RCTs) at 3 months (19.0–94.1%), 4 months (45.9–91.9%), 6 months (3.0–88.0%), and 12 months (37.0–90.7%) [16]. Similarly, non-RCT studies also found a wide range of retention at 3 (68.0–87.0%), 6 (21.4–78.1%), or 12 (26.0–85.0%) months [16]. Median retention rate across all studies has been found to be approximately 58% at 12 months and 38.4% at three years, with methadone cohorts reporting higher median retention rate at 6 and 12 months compared to buprenorphine cohorts and mixed OAT cohorts [17]. This is consistent with previous studies suggesting that buprenorphine is associated with shorter duration of treatment relative to methadone [17, 18]. In a meta-analysis comparing methadone to slow-release oral morphine (SROM), the difference in dropouts was not statistically significant between participants receiving either medication [19].

Along with type of substitution medication, factors related to the dose, prior treatment experience, and treatment readiness have all been associated with an individual’s treatment retention [17, 20–23]. Similarly, sociodemographic factors such as age, gender, marital status, employment, ethnicity, as well as health-related factors such as physical health, mental health, addiction severity, suicide attempts and polysubstance have been found to contribute to retention in treatment [17, 20, 21, 23–27]. For instance, younger age, substance use, lower doses, criminal activity, and negative attitudes towards treatment appear to be associated with reduced retention in OAT [17].

OAT was officially implemented in Austria in 1987 [28]. In the beginning, access to OAT was quite limited (e.g. failed trials of abstinence orientated treatment, HIV infection, pregnancy) but these restrictions have since disappeared, and OAT has become the most important treatment option for opioid dependence in Austria [28]. It is estimated that between 50 and 60% of individuals with opioid use disorder are in OAT in 2019 [29]. Methadone was originally the only substitution medication available, but SROM was added in 1998, buprenorphine in 1999 and buprenorphine/naloxone in 2008 [28]. In 2018, 55% of patients were treated with SROM, 19% with buprenorphine, 11% with methadone, and 13% with methadone/levomethadone [29]. Almost all OAT medications are covered by health insurances and can be initiated by general practitioners who have completed a special training and who continue to attend regular trainings to keep up-to-date [30]. General practitioners play an important role in OAT in Austria: 73% of OAT patients were treated by general practitioners in 2019 [29]. Other licenses also exist to allow other general practitioners to maintain OAT, but without the ability to change the dosage or medication [30]. Prescription of substitution medication is controlled nationally, and the medication is dispensed in pharmacies on a daily basis and consumed under observation by the pharmacists. There are exceptions however, such as take-home doses for the holidays as well as for patients who are very well integrated and stable. All substitution prescriptions are regulated by the Austrian Narcotics Act [31] and related decrees [32].

The Austrian substitution registry was set up and has been maintained by the Federal Ministry of Health since 1989, when opioid agonist treatment was officially implemented. Physicians were asked to notify the registry about the beginning and end of each of their patient's substitution treatment in order to prevent patients from being treated by more than one physician at the same time. At the beginning, the quality of the data was limited due to the fact that the completeness of the registry could not be assumed. In particular, the registry was not systematically informed about a certain percentage of treatment terminations, resulting in an increasing number of “ghost cases” in the registry. These were generated when patients did not show up to appointments, and therefore did not provide physician with a formal treatment termination. An important step to improve data quality was made in 2008, when public health officers supervising every substitution treatment became involved in the registration procedure by supervising every OAT prescription. Public health officers were required to validate each long-term OAT prescription at least monthly. At the same time, the Federal Ministry in Austria started quality checks of the data and systematically eliminating ghost cases.

At the beginning of 2011, a new online-based system called “eSuchmittel” was introduced in order to assure completeness and quality of the registry. Since then, each patient entered in the OAT registry is identified via the central population registry, preventing double entries. In the process of setting up eSuchtmittel, all existing entries were systematically quality checked and, if necessary, corrected. In addition, several tools and mechanisms have been implemented to ensure continuous data quality. The data are pseudonymized for statistical analysis, while still including relevant information such as year of birth, sex, substitution medication, initial dose prescribed and region.

The objective of this study was to analyze the retention rates in the Austrian OAT system, to explore the differences between the various substitution medications, and to identify patient characteristics associated with retention, all using data collected by the newly implemented Austrian registry.

## Methods

### Study sample

For the present analysis, data from all patients starting OAT between 01/01/2011 and 31/12/2012, and therefore registered in the new online-based system called eSuchmittel, were used, and this cohort was followed until 30/04/2013. The data used for this analysis includes 4,778 patients for a total of 5,165 treatment sequences. Each treatment sequence represents an independent OAT episode that was started within the analysis period by a patient registered in the registry and that was ended due to treatment discontinuation or due to study completion (end of analysis period). If the registry revealed two treatment episodes for the same patient which were no more than one month apart, these episodes were aggregated into one single treatment sequence. Treatment was actually uninterrupted for these sequences, these artificial separations appear in the registry if patients moved to another region, changed physicians or institution, etc. This is common procedure in studies using registry data [11]. If patients started a new treatment more than one month later, this was treated as a new independent sequence. The overwhelming majority of patients had only a single treatment sequence (92.8%; Table 1).

**Table 1:** Characteristics of all 4778 patients in the cohort

Characteristics		
OAT sequences		
Patients with 1 sequence	92.8%	
Patients with 2 sequences	6.4%	
Patients with 3 sequences	0.7%	
Patients with 4 sequences	0.08%	
Sex (%)		
Male	75.0%	
Female	25.0%	
Age when treatment started		
< 20	4.8%	
20–24	25.7%	
25–29	27.9%	
30–39	27.2%	
> 39	14.4%	
Region		
Vienna	28%	
Other Austrian federal states	72%	

Substitution medication	Start of sequence	End of sequence
Methadone	23.2%	20.5%
Levo-methadone	6.9%	8.4%
Buprenorphine	25.9%	24.3%
Buprenorphine/naloxone	5.7%	5.0%
Slow-release morphine	36.2%	39.9%
Other	1.1%	1.4%
Unknown	1.0%	0.5%

### Measures

From the administrative records of patients accessing opioid substitution treatment in Austria between January 2011 and December 2012, demographic characteristics such as sex, age, as well as region and substitution medication used were extracted to assess their association with treatment retention. All the data used in this study were inputted into the system by public health officers at the start of treatment and kept up to date at each follow-up. For the analysis of regional treatment, the most recent information was used (Additional file 1: Table 1). For example, if the patient moved to Vienna from another region, the patient was counted as a Viennese patient. Moreover, for each patient, the most current medication enlisted in the system was used to determine the appropriate medication cohort. For instance, if the patient´s medication changed from buprenorphine to methadone, the client was assigned to the methadone group. Of note, changes in medication were rare in the cohort—only 16% of the treatment sequences reporting a change. The proportion of substitution medication at the start of treatment sequences are almost the same as at the end of treatment sequences (Table 1).

### Statistical analysis

Since the observation times were different for the individuals entering OAT, ranging between 120 and 851 days, an analysis method was needed to adequately deal with censored data. The database holds no left censored data since only patients that entered during the observation period were included, whereas patients already in treatment when the observation period started were not included. However, the database has right censored data since patients may die or move away and/or stop treatment without notifying public health officers. Kaplan–Meier survival and Cox regression analyses were used to compensate for right censorship as these are adequate methods to estimate cumulative dropout risk at different follow-up times [33]. The Tarone-Ware test was used in conjunction with the Kaplan–Meier curves; a multivariate Cox model was calculated using the variables available, namely age, sex and substitution medication. The database holds no information on illicit opioid of choice, history of injection drug use, education and socioeconomic status, so these could not be added to the model. These analyses were performed with the SPSS 17.0 program package [34].

### Ethics and study design

All data are collected according to the Federal Austrian Narcotic Act, which explicitly states that scientific analyses with these data may be performed on behalf of the Ministry of Health. Moreover, the Austrian Public Health Institute is owned by the Austrian Ministry of Health and regulated by federal law. The Austrian Public Health Institute performs statistical analyses for the Ministry of Health. This study was therefore carried out by the Austrian Public Health Institute and was approved by the Austrian Ministry of Health. This study is reported following the Strengthening the Reporting of Observational Studies in Epidemiology (STROBE) guidelines [35].

## Results

The characteristics of the patients entered into the study are documented in Table 1. Overall, 4,778 patients were included, the majority of which were male (75%). Patients age at the beginning of treatment seemed mostly split between the following 3 age groups: 20–24 years old (25.7%), 25–29 years old (27.9%) and 30–39 years old (27.2%). At the most recent time point, 28% of patients were located in Vienna and the rest in other Austrian federal states. Slowrelease morphine, buprenorphine, and methadone represent the 3 most common substitution medication within the Austrian treatment system.

### Differences in sex

**Fig. 1:**
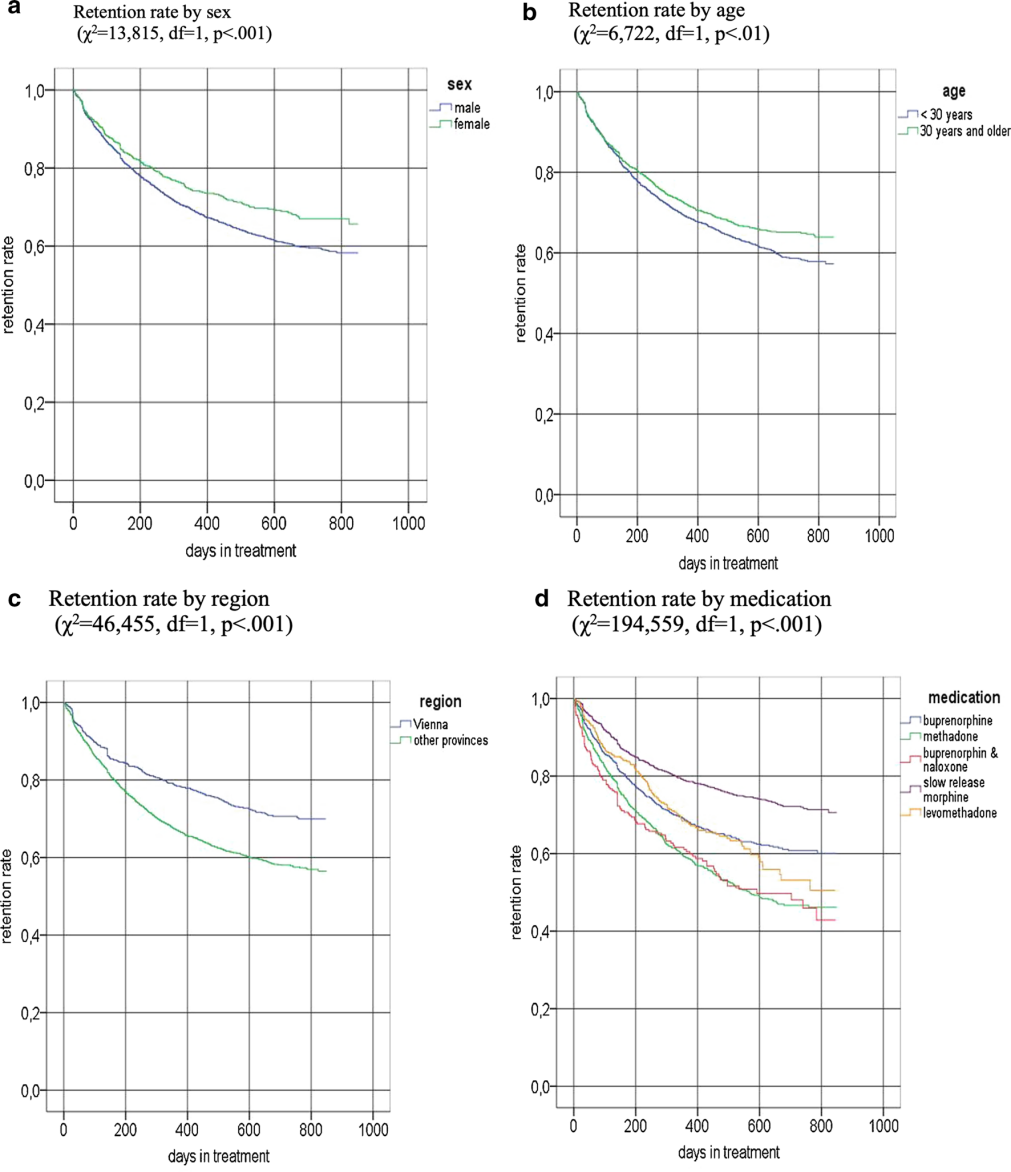
Kaplan–Meier curves of retention in treatment by sex (**a**), age (**b**), region (**c**) and substitution medication (**d**), in conjunction with Tarone–Ware test

The retention rates between males and females were significantly different, as demonstrated by the Kaplan–Meier curves (χ^2^ = 13,815, df = 1, p < 0.001; Fig. 1). Male patients had a significantly lower retention rate when compared to female patients, as demonstrated by the multivariate Cox regression model (exp(B) = 0.806, 95% CI 0.714–0.908; Table 2).

**Table 2:** Multivariate Cox regression model of retention in treatment by sex, age, region and substitution medications

Variables	*B*	SE	Wald	*df*	Significance	Exp(*B*)	95% CI Exp(*B*)
Sex (male)	− .216	.061	12.443	1	.000	.806	0.714–0.908
Age (older than 30)	.144	.052	7.799	1	.005	1.155	1.044–1.279
Region (Vienna)	.364	.062	33.918	1	.000	1.439	1.273–1.626
Buprenorphine	.332	.068	24.190	1	.000	1.394	1.221–1.591
Buprenorphine/naloxone	− .037	.105	.126	1	.723	.964	0.785–1.183
Slow-release morphine	.761	.065	137.959	1	.000	2.141	1.885–2.430
Levo-methadone	.339	.095	12.629	1	.000	1.404	1.164–1.693

### Differences in age

In Austria, the mean age of patients first entering treatment for heroin use is 29 years old [36]. By using 30 years of age for simplicity, patients were grouped as either "above 30 years old" or "below 30 years old. This dichotomous variable was then assessed based on retention. Analysis of the survival curves demonstrated significant differences between retention rates (χ2 = 6,722, df = 1, p < 0.01; Fig. 1). The Cox regression revealed that older patients were more likely to be retained than younger patients (exp(B) = 1.155, 95% CI 1.044–1.279; Table 2).

### Regional differences

Since Vienna is the only metropolitan city in Austria, it makes sense to compare Vienna with the rest of Austria. The retention rate of individuals living in Vienna was significantly different to those living in other parts of Austria (χ^2^ = 46,455, df = 1, p < 0.001; Fig. 1). From the Cox regression model, those living in Vienna has a significantly higher retention rate (exp(B) = 1.439, 95% CI 1.273–1.626; Table 2).

### Differences in medication used

From the Kaplan–Meier curves, major differences in retention rates based on substitution medication can be observed. The Tarone-Ware test demonstrate these differences are significant (χ^2^ = 194,559, df = 1, p < 0.001; Fig. 1). The retention rate was significantly higher when slow-release morphine was prescribed (exp(B) = 2.141, 95% CI = 1.885–2.430; Table 2). The two-year retention rate was lowest for methadone (47%) and buprenorphine/naloxone (48%), and noticeably higher for slow-release morphine (71%) (Table 3).

**Table 3:** Retention rates by substitution medication at 6 months, 1 year, and 2 years

Substitution medication	6 months (%)	1 year (%)	2 years (%)
Methadone	71	59	47
Levo-methadone	82	67	50
Buprenorphine	78	68	59
Buprenorphine/naloxone	68	59	48
Slow-release morphine	86	79	71
All medications	80	70	61

## Discussion

This study aimed to analyze retention rates within the Austrian substitution treatment system using data collected by the newly implemented Austrian registry. By analyzing administrative records from 4,778 patients accessing opioid substitution treatment between January 2011 and December 2012, patient characteristics associated with retention were identified. Firstly, women had significantly higher retention rates than men, which is in-line with findings in other studies [11, 21, 37]. Secondly, individuals who were older had significantly higher retention rates, which is also in line with a number of studies that have found older patients to be more stable and better supported than younger patients [11, 26, 38–42]. Thirdly, individuals living in Vienna have a significantly higher retention rate, in part due to the higher density of physicians well-versed in OAT as well as the shorter geographical distance between patient and treating physician, making it easier for the patients to uphold daily administration schemes [26, 43]. Lastly, different substitution medications were associated with significantly different retention rates, with SROM far exceeding all other medications [44, 45].

The Austrian one-year retention rate of 70% is among the highest retentions rates when compared to other published studies: 35.5% in British Columbia, Canada [46], 50% in Washington, United States [47], 61% in Ireland [11], 58% in Baltimore, United States [21], 65.8% in Ukraine [48], 73.9% in Tel-Aviv [49], 62% in Las Vegas, United States [40], 53.9% in China [26], 72.5% in Norway [50], and 60.4% in Germany [51]. In a systematic review including 63 observational cohort studies reporting on retention rates, the median retention rate was approximately 57% at 12 months [17]. Caution is needed when comparing these retention rates however, since many factors can influence retention. Treatment programs and treatment guidelines, as well as cost and distribution models for the medications are all important in providing context when assessing retention rates between countries. OAT is the first-line treatment for opioid dependence in Austria, and facilities are generally low threshold access. Moreover, Austria is a country in which a variety of opioids is available for OAT, which is rare in an international context, and the decision to start substitution treatment depends largely upon patients and providers, unaffected by financial, regulatory, attitudinal, and logistic constraints [52–54].

The most important finding of this analysis is the significantly higher retention rate of SROM when compared to other forms of OAT. The clinical utility of SROM for opioid dependence has been reported previously and SROM is successfully used in Austria, Slovenia, Bulgaria and Switzerland [44, 55, 56]. Formulated to deliver morphine at a controlled dose over 24 h, it is similar to other long-acting mu-opioid receptor agonists such as methadone, but has fewer cardiotoxicities, drug-drug interactions, and can be titrated more rapidly [56, 57]. A recent meta-analysis showed that SROM is equivalent to methadone in retaining patients in treatment and reducing illicit heroin use, while potentially reducing cravings [19]. SROM was also associated with higher treatment satisfaction and lower mental stress, depression, and anxiety in some studies, but with mixed evidence on quality-of life improvements compared to treatment with methadone or buprenorphine [19, 56, 58–60]. Additionally, maintenance treatment with SROM appears to be a clinically useful alternative treatment in patients not tolerating methadone or with inadequate withdrawal suppression [44, 61, 62]. The 2018 Canadian national clinical practice guidelines include SROM as a third-line option for patients with Opioid Use Disorder (OUD) refractory to buprenorphine and methadone [63]. In Switzerland, while the use of diacetylmorphine (approved in 1994) and buprenorphine (approved in 2002) seems to have reached a ceiling, SROM (approved in 2013) is expected to double or triple in use over the next few years [64]. As demonstrated by the Austrian registry data, SROM is broadly used in Austria and its higher retention suggests it can be effective in treatment. Of note, SROM is more expensive than methadone in Austria but substitution medication is covered by health insurance costs and therefore does not play a role at individual level. More thorough research is needed on the effectiveness of SROM in the treatment of OUD. As the majority of SROM trials are small and unblinded, further studies are necessary [65].

The present study found a 6-month buprenorphine/naloxone retention rate of 68% and a 12-month retention rate of 59%. This is higher than what was found in a prospective, open-label, multicenter study using buprenorphine/naloxone (n = 307) conducted from April 2008 to August 2011 in Austria, which resulted in a 6-month retention rate of 57% and a 12-months retention rate of 46% [66]. Buprenorphine/naloxone's lower retention rate over a decade ago is likely due to its novelty in Austria at the time, which was only approved in 2008 [28, 66]. Moreover, the development of more convenient induction methods has helped reduced barriers and expand buprenorphine/naloxone use [64, 67–69].

The in-depth overview of a complex system of care like the Austrian substitution treatment system performed in this study requires specific tools such as an online-based registry, which is highly regulated for various reasons. Most countries globally do not have such tools implemented or are not using them. Such a systematic overview is necessary to inform and control the quality of care, costs and outcomes of a system. The simple counting of adverse events such as overdose cases is not sufficient; collecting treatment data and specific outcome indicators is much more effective. The implementation of the Austrian substitution treatment registry has allowed for the systematic evaluation of treatment retention and quality of care on an individual and system-wide level.

Using registry data to analyse retention rates or different outcomes of substitution treatment is an established method in some countries [4, 37, 70, 71]. It has to be kept in mind that the primary purpose of creating such a registry and supplying data to the registry is administrative. Data quality, especially with regards to “ghost cases” and unique identification, is a concern when using registry data. But often, valuable and important data that are collected by registries are not used and analyzed due to a lack of capacity or expertise. Their results are therefore not published on a regular basis and cannot provide up-to-date information to providers. Resistance from health professionals, who often deem data collection as an additional workload, makes such solutions difficult to implement. However, this worked relatively well in Austria because of the simplicity and ease of the data collection using the online approach. When the Austrian substitution treatment system was migrated into an online registry (eSuchtmittel), efforts were made to ensure that the quality of the data would be sufficient enough to allow it to be used for epidemiological purposes and be extrapolated to improve patient outcomes, inform OAT services, and better manage OAT delivery. As an example of a similar system-wide approach, the Irish drug treatment services has recently developed a remote model of care using the national healthcare system for the continued assessment and ongoing provision of care to OAT patients while mitigating the risks associated with COVID-19 [72].

A main strength of this analysis is the naturalistic design of the study. A prospective naturalistic study design evaluates patients in a natural setting over time, with no attempts at intervention, thereby increasing the generalizability and external validity of the research findings. This study design has been successfully applied in previous research, including a similar study done using OAT registries in Switzerland [64]. Another strength of this analysis is its large sample size. Since all Austrian patients registered in the opioid substitution treatment are included, there was no risk of selection bias.

### Limitations

Several limitations need to be considered when interpreting our data. By virtue of its naturalistic design, unlike more monitored and restricted research settings, important cofounders such as baseline substance use patterns, preferred route of substance use, co-morbid substance use patterns, medication dose, reason for treatment termination, socioeconomic indicators were unmeasured and not included in our regression model. Moreover, our analyses only used retention as the primary outcome and did not to take into account other markers of treatment success. Indeed, outcomes such as overdose, illicit drug use, abstinence are important to include when evaluating the overall quality of substitution treatment systems. In addition, we did not perform statistical analysis to account for potentially correlated outcomes within-individual, though only 8% of patients had multiple treatment sequences during the study period. Finally, we treated all variables as time-invariant by using the last region and last medication listed in the database. Though changes in region and medication were rare (only 16% of treatment sequences reported a change in substitution treatment), we did not perform any sensitivity analyses censoring data from patients who changed region or substitution medication during the treatment sequence.

### Conclusion

The Austrian substitution registry provides a useful resource with high external validity. Though it only includes a limited set of variables, it allowed for the systematic evaluation of OAT in Austria and the significance of specific factors on treatment retention. Though difficult to compare with other studies because of the varying methodologies, the retention rate in the Austrian substitution treatment system is quite high relative to other countries. One of the most striking results of the present study is the significant superiority of SROM in terms of the treatment retention. This is encouraging data for SROM's clinical effectiveness but other aspects need to be considered and should be thoroughly researched. Developing registries similar to the one implemented in Austria would enable rapid data collection and efficient large-scale comparisons which could be analysed to improve patient outcomes.

## Supplementary information

**Additional file 1:** **Table 1.** Substitution medication by region.

**Additional file 2:** STROBE Checklist..

## Data Availability

The datasets used during the current study were collected by the Austrian substitution registry and maintained by the Federal Ministry of Health. As such, restrictions apply to the availability of these data, which were used under license for the current study and which are not publicly available. Data are however available from the authors upon reasonable request and with permission of Federal Ministry of Health.

## Abbreviations

OAT: Opioid agonist treatment
OUD: Opioid use disorder
SROM: Slow-release oral morphine
