# Supplementary material for: Retention in the Austrian opioid agonist treatment system: a national prospective cohort study
Source: Harm Reduct J. 2021 Feb 24;18:25. doi: 10.1186/s12954-021-00473-9 (PMC7903033; doi:10.1186/s12954-021-00473-9)
Supplement: Supplementary file 1 — Additional file 1: Table 1. Substitution medication by region. [file 12954_2021_473_MOESM1_ESM.pdf]

Additional File 1. Substitution medication by region

Table 1: Proportion of substitution medication by region

Substitution Medication	Vienna	Other Regions
Methadone	14.5%	23.3%
Levo-methadone	6.6%	8.6%
Buprenorphine	24.3%	23.8%
Buprenorphine/naloxone	5.2%	5.3%
Slow release morphine	48.2%	36.3%
Other	0.1%	1.9%
Unknown	1.1%	0.9%
